# Genetic Networks Controlling Structural Outcome of Glucosinolate Activation across Development

**DOI:** 10.1371/journal.pgen.1000234

**Published:** 2008-10-24

**Authors:** Adam M. Wentzell, Ian Boeye, Zhiyong Zhang, Daniel J. Kliebenstein

**Affiliations:** 1 Genetics Graduate Group, University of California Davis, Davis, California, United States of America; 2 Department of Plant Sciences, University of California Davis, Davis, California, United States of America; The University of North Carolina at Chapel Hill, United States of America

## Abstract

Most phenotypic variation present in natural populations is under polygenic control, largely determined by genetic variation at quantitative trait loci (QTLs). These genetic loci frequently interact with the environment, development, and each other, yet the importance of these interactions on the underlying genetic architecture of quantitative traits is not well characterized. To better study how epistasis and development may influence quantitative traits, we studied genetic variation in Arabidopsis glucosinolate activation using the moderately sized Bayreuth×Shahdara recombinant inbred population, in terms of number of lines. We identified QTLs for glucosinolate activation at three different developmental stages. Numerous QTLs showed developmental dependency, as well as a large epistatic network, centered on the previously cloned large-effect glucosinolate activation QTL, *ESP*. Analysis of Heterogeneous Inbred Families validated seven loci and all of the QTL×DPG (days post-germination) interactions tested, but was complicated by the extensive epistasis. A comparison of transcript accumulation data within 211 of these RILs showed an extensive overlap of gene expression QTLs for structural specifiers and their homologs with the identified glucosinolate activation loci. Finally, we were able to show that two of the QTLs are the result of whole-genome duplications of a glucosinolate activation gene cluster. These data reveal complex age-dependent regulation of structural outcomes and suggest that transcriptional regulation is associated with a significant portion of the underlying ontogenic variation and epistatic interactions in glucosinolate activation.

## Author Summary

A principal interest in biology is to understand how natural genetic variation translates into phenotypic variation. A key component of this connection is how the genetic variation interacts with other sources of variation, such as environment (G×E), development (G×D), or other genetic loci (G×G or epistasis). To analyze the molecular underpinnings of these quantitative genetics interaction terms, we investigated the genetic architecture of an adaptive trait, glucosinolate activation, in *Arabidopsis thaliana* during the development of what is considered a static mature rosette. Variation in glucosinolate activation was principally controlled by epistatic and G×D interactions. Epistatic interactions identified both Mendelian epistasis, where regulatory loci controlled enzymatic loci, and quantitative interactions between regulatory loci. G×D appeared to involve master regulatory loci as determined by *trans*-eQTL hotspot analysis. Finally, two common glucosinolate activation QTLs appear to have evolved via gene loss and sub-functionalization following quadruplication of an ancestral genomic fragment, potentially by two whole-genome duplications. Thus, genomic duplication events may facilitate the formation of quantitative genetic variation. This study provides insights into the molecular basis of the link between genetic and phenotypic variation in a potentially adaptive trait.

## Introduction

Most phenotypic variation present in natural populations is under polygenic control, largely determined by genetic variation at multiple quantitative trait loci (QTLs), which has motivated considerable efforts to elucidate the genetic basis of these polygenic traits [1]–[3]. A complete understanding of quantitative traits necessitates identification of the underlying genes and their associated additive, dominance, and epistatic effects [2]. In addition, the underlying genetic architecture of many quantitative traits may vary across development and different environments. As such, a comprehensive description of a quantitative traits genetic architecture requires analysis in several developmental or environmental contexts to assess stability of the genetic architecture [2],[4],[5].

QTL mapping, which measure the association of genetic markers with phenotypic variation, is one of the most common approaches for identifying loci and epistatic interactions controlling polygenic inheritance [1]. Improved statistical models, marker technology, and genomic resources have facilitated QTL mapping experiments for a wide array of quantitative traits, ranging from development and morphology to metabolism and disease resistance [2],[4],[5]; However, QTL mapping experiments are often limited to a single stage in development and one or few environments. As a consequence, there is little information available to answer the question of how the underlying genetic architecture varies across developmental and environmental contexts.

Accurate characterization of a quantitative trait's underlying genetic architecture is often limited by practical considerations that limit the number of progeny included in a mapping analysis. Small populations are especially problematic in the presence of epistasis between QTLs, as the pair wise comparisons required to detect these interactions rapidly exhausts the available genotypic variation, leading to an underestimation of numbers of loci and interactions, resulting in an incomplete picture of the genetic architecture [6],[7]. One common type of epistasis occurs when a trait is controlled by one or few large effect loci and numerous modifier QTLs of smaller effect, a situation frequently observed in plant disease resistance [8]–[16]. In such systems, the effects of any modifiers are most detectable in those lines containing the appropriate allele at the large effect locus; however this reduces by half the population in which to detect these smaller effect loci, significantly reducing statistical power [5]–[7]. Thus, resolution of the underlying genetic basis of complex traits requires the analysis of large populations across different environments or developmental stages [17],[18].

**Figure 1:**
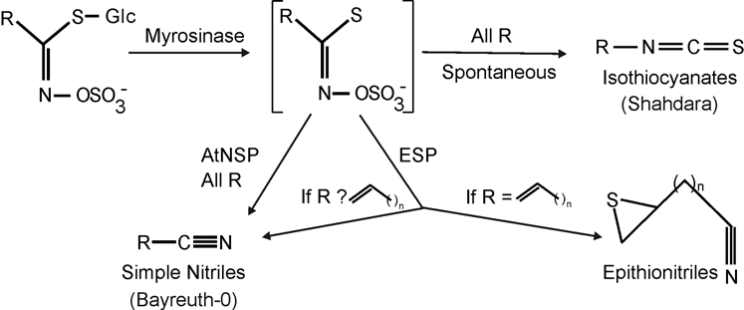
Glucosinolate Activation and the Subsequent Structural Rearrangement. Myrosinase enzymes initiate glucosinolate activation by hydrolyzing the thioglucose bond, generating an unstable aglycone intermediate (bracketed). This intermediate can spontaneously rearrange into the isothiocyanate structure. Epithionitrile structural specifiers such as *ESP* can promote the formation of epithionitrile structures from glucosinolates with a terminal double bond, and simple nitriles from all other glucosinolates. As yet unidentified Arabidopsis simple nitrile structural specifiers (AtNSPs) promote the formation of simple nitrile structures from all glucosinolates. In parenthesis are the Bayreuth and Shahdara accession showing their predominant glucosinolate activation form.

To investigate how development and epistasis can interact to control the variation in an adaptive trait, we studied the outcome of glucosinolate activation within *Arabidopsis thaliana* using a moderately sized recombinant inbred population. Glucosinolates are the inert storage form of a two part phytochemical defense system found throughout the *Brassicaceae*, where biologically active structures are catabolically produced by the enzyme myrosinase [19]–[21](Figure 1) . The particular structural outcome, as defined by the chemical structure of the end product, of glucosinolate activation plays an important role in plant defense against insect herbivory [22]–[25], as well as the nutritional and flavor characteristics of brassicaceous crops [26]. Further, the structural outcome shows significant intraspecific diversity such that natural accessions activate a glucosinolate to either a nitrile or isothiocyanate depending upon their genotype. Thus an improved understanding of the genetic basis of variation in structural outcomes has important potential implications in evolution and ecology as well as nutrition and agriculture.

Glucosinolate activation in Arabidopsis provides an excellent model for studying how development and epistasis influence quantitative traits, with a molecularly characterized biochemical pathway comprising demonstrated epistatic interactions and developmental variation. During glucosinolate activation, the myrosinase enzyme catabolically generates the unstable intermediate. The final structural outcome of subsequent rearrangement of this unstable intermediate is influenced by the presence or absence of various structural specifier proteins (Figure 1). The *Epithiospecifier Protein* (*ESP*) and an as yet unidentified simple nitrile structural specifier (*AtNSP*), promote the formation of simple and epithionitriles at the expense of the default isothiocyanate rearrangement via two biochemically related yet separate rearrangements (Figure 1) [22], [23], [27]–[31]. The *Epithiospecifier Modifier* (*ESM1*) epistatically modulates *ESP* mediated epithionitrile and simple nitrile rearrangements (Figure 1)[22],[23]. The observation of quantitative variation influencing the developmental regulation of glucosinolate activation enabled us to explore the stability of QTLs and epistatic interactions across development [30],[32].

We used the Bayreuth (Bay-0)×Shahdara (Sha) recombinant inbred lines (RILs) [33] to map QTLs controlling the structural outcome of glucosinolate activation in Arabidopsis. These parental accessions contain genetic variation for *ESP* and *ESM1* and differ in developmental regulation of structural outcomes [32]. We measured structural outcomes at 30, 35 and 42 days post germination (DPG), and compared the resulting maps to assess the stability of the underlying genetic architecture across development. These DPG were chosen because day 30 represents the end of logarithmic growth in all of the lines, or Stage 1.10, while day 42 is one week away from the earliest RIL flowering (Stage 5.10) in our environmental conditions [32],[34],[35]. Thus, we can focus on developmental changes in what is typically considered a static rosette and is also the tissue and stages where lepidopteran insects predominate on Arabidopsis in the wild [36]. This analysis identified eleven loci and twelve pair wise epistatic interactions influencing structural outcomes, as well as eight different QTL×DPG interactions. Heterogeneous Inbred Families (HIFs) differing only for their genotypes at each QTL locus validated seven loci and all of the QTL×DPG interactions tested. The availability of transcript accumulation data within 211 of these RILs [37] enabled comparison of expression QTLs (eQTLs) for structural specifier genes and their homologs, which demonstrated collocation of eQTL clusters with eleven of the identified loci. These data reveal complex age dependent regulation of structural outcomes and suggest that transcriptional regulation is associated with a significant portion of the underlying variation, and may explain the epistatic interactions described here.

## Results

### Structural Outcomes of Glucosinolate Activation in Bay-0 and Sha

**Figure 2:**
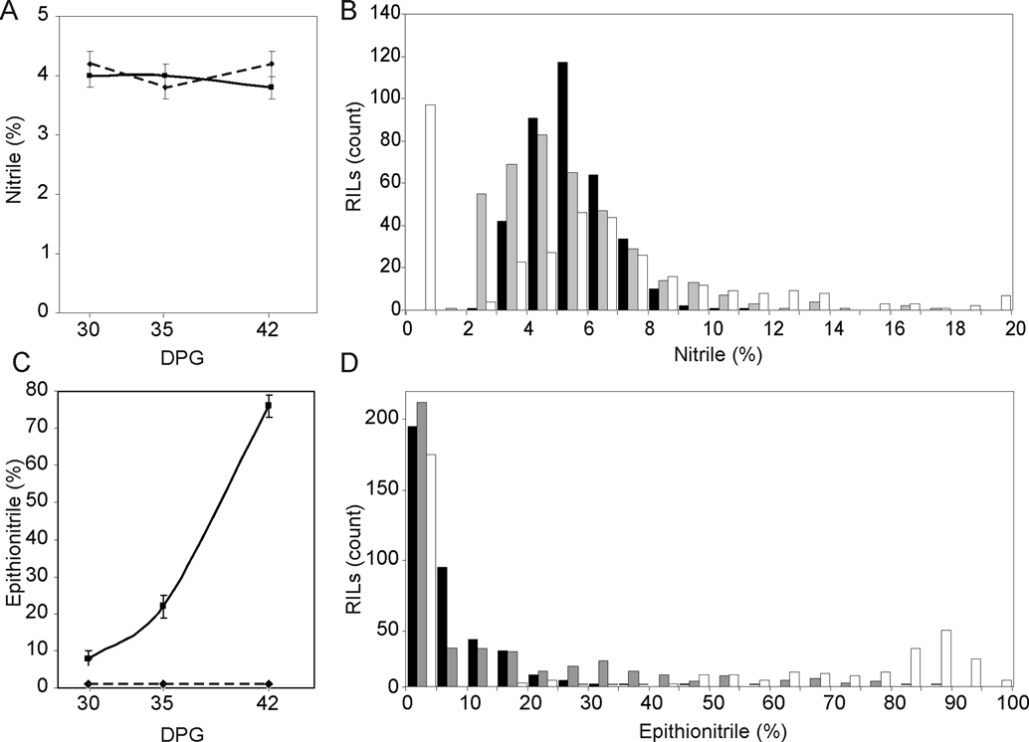
Ontogenic variation of Structural Outcome in Glucosinolate Activation. GC-FID was used to measure the structural outcome from exogenous allyl glucosinolate activation in the RILs and interplanted Bay-0 (dashed line) and Sha (solid line) parental accessions at 30, 35, and 42 DPG. For the parental analysis, averages with standard errors are shown. For the RILs, The percent simple nitriles were binned into 1 percent intervals from 0 to 20 percent, and percent epithionitriles were binned in 5 percent intervals from 0 to 100 percent and the number of RILs per bin are shown. For the RILs, black bars show the distribution as measured at 30 DPG, grey is 35 DPG and white at 42 DPG. A) Simple nitriles in Bay-0 (dashed) and Sha (solid) parental accessions at 30, 35, and 42 DPG. B) Distribution of simple nitrile formation in the RILs. C) Epithionitrile formation in parental accessions. D) Distribution of epithionitrile formation in the RILs.

Bay-0 and Sha contain different glucosinolates due to variation at the *GSL-AOP* and *GSL-Elong* loci, such that Bay-0 has 3-hydroxypropyl glucosinolate as its main short chain aliphatic glucosinolate and Sha has but-3-enyl glucosinolate [38]. These two accessions also differ in the structures they produce following activation of these glucosinolates. Bay-0 lacks functional *ESP* and produces mixtures of simple nitriles and isothiocyanates, depending on the age of the plant [32]. In contrast Sha possesses a functional allele of *ESP* and produces mixtures of epithionitriles, simple nitriles and isothiocyanates (Figure 1). In agreement with previously published analysis, interplanted Sha parental controls had an increasing epithionitrile proportion during development for both the exogenous (Figure 2A and C) and endogenous glucosinolate substrates (Table S4)[32]. In contrast to Sha, the Bay-0 parent showed little variation in the structural outcome of exogenous allyl glucosinolate activation between 30, 35 and 42 DPG (Figure 2A and C). The activation products for the endogenous 3-hydroxypropyl glucosinolate produced in Bay-0 could not be detected in this experiment. Thus, there is developmental variation in glucosinolate activation between the Bay-0 and Sha parental accession which allows us to investigate how the genetics of glucosinolate activation interact with plant development.

### Distribution of Structural Outcomes in the RIL Population

We measured the structural outcome of glucosinolate activation using exogenous allyl glucosinolate in the Bay-0×Sha RILs and compared the trait distribution to the interplanted parental controls. The use of exogenous allyl glucosinolate allowed us to mask effects of variation in glucosinolate biosynthesis and accumulation. Considerable transgressive segregation was observed for simple nitrile where there were numerous RILs with values higher and lower than the Bay-0 or Sha parents (Figure 2B). Further, there was transgressive segregation for epithionitrile as evidenced by the RILs with a higher value than the Sha parent (Figure 2D). This suggests that alleles promoting the formation of each structure exist in both parents. This is particularly surprising in the case of epithionitrile proportions, as the Bay-0 parent can not produce epithionitriles due to a lack of functional *ESP* and as such might not be expected to contain genes enhancing epithionitrile formation.

This population includes 212 lines producing no epithionitrile structures following activation of allyl glucosinolate (Figure 2D). This is consistent with the previously observed requirement of a functional *ESP* allele for epithionitrile production [27],[30],[32]. For the sub-population of RILs with a functional allele of *ESP*, the distribution gradually shifted towards increased epithionitrile production from 30 DPG to 42DPG, in a manner consistent with the epithionitrile increase observed in the Sha parental controls (Figure 2C). However, individual RILs showed possible differences in the both the direction of and magnitude of change in epithionitrile production from 30 to 42 DPG, suggesting that there is genetic variation in age dependent control of epithionitrile proportion from 30 to 42 DPG (Table S4). These possible differences will however require identification of the QTL and HIF validation to ensure that this is not random variation around the mean.

The distribution of nitrile formation within the RILs did not show a similar ontogenic shift, but there were numerous lines that had no simple nitrile formation at 42 DPG (Figure 2B). This low or undetectable amount of simple nitriles is connected to epithionitrile proportions approaching complete utilization of the glucosinolate substrate in these RILs at this DPG. This difference in ontogenic regulation supports the model of simple nitrile and epithionitrile production as being independent processes competing for the same substrate.

### Heritability of Structural Outcomes

To compare the underlying genetics controlling the biochemically distinct nitrile and epithionitrile glucosinolate activation outcomes, we estimated heritability for each glucosinolate activation structure from each measured glucosinolate (Table 1). Because we were spatially constrained to only a single measurement of each RIL at each DPG, this heritability estimate includes both RIL and RIL×DPG effects and environmental variance is not perfectly controlled. The endogenous glucosinolates are each limited to roughly one quarter of the RILs due to independent assortment of the *GSL.AOP* and *GSL.Elong* biosynthetic loci [38]. Further, the 4-methylsulfinylbutyl glucosinolate which derives from transgressive segregation at the *GSL.AOP* and *GSL.Elong* lacks a terminal alkene functional group, and can only form simple nitrile and isothiocyanate structures (Figure 1)[39],[40]. For all detectable exogenous and endogenous glucosinolates, the heritability of simple nitrile and epithionitrile proportions was approximately 50%, with the sole exception of epithionitrile from but-3-enyl glucosinolate at 69%.

**Table 1:** Heritability of Structural Outcomes of Glucosinolate Activation.

Trait	*P* Value		Type III Sums of Squares			Heritability
	Geno	DPG	Model	Geno	DPG	
% Simple Nitriles	<0.001	0.397	13083.4	6603.8	17.1	50.5
% Epithionitriles	<0.001	<0.001	874209.6	427015.0	166069.7	48.8
Butenyl % Simple Nitriles	<0.001	<0.001	54039.5	28550.7	19106.5	52.8
Butenyl % Epithionitriles	<0.001	<0.001	124753.5	82196.3	15517.3	65.9
4MSO % Simple Nitrile	<0.001	<0.001	348841.6	178484.9	58429.9	51.2

Geno represents the genotype term in the model. DPG is the days-post-germination factor in the model.

### Structural Proportion QTLs

**Figure 3:**
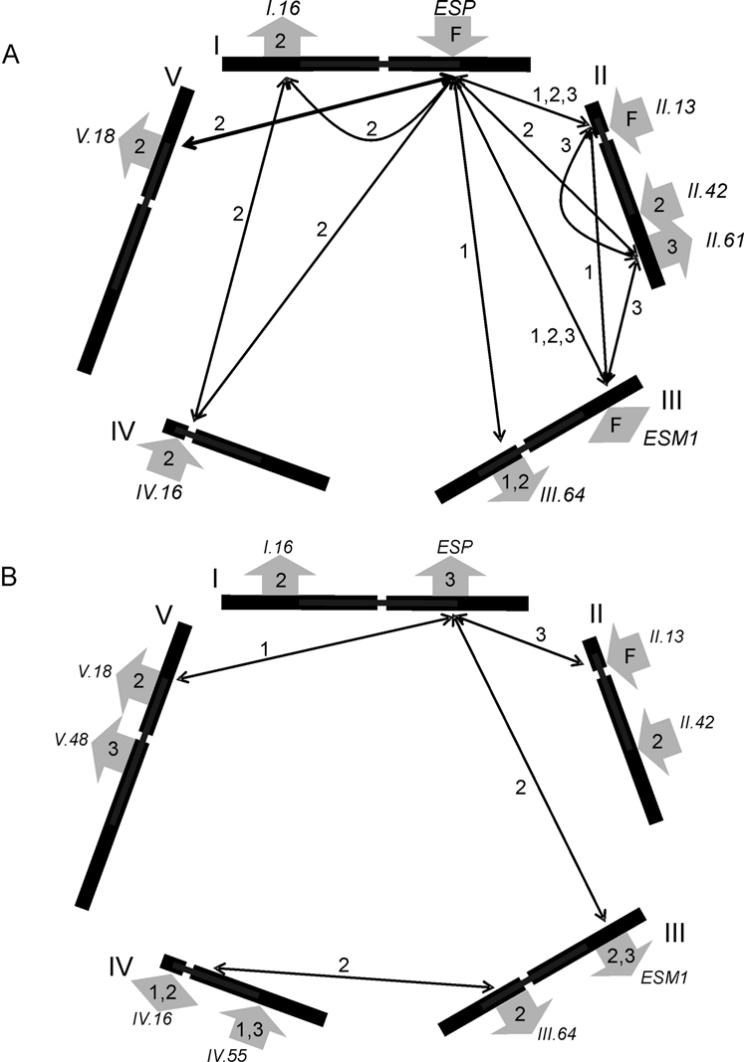
QTLs Controlling the Structural Outcome of Glucosinolate Activation. The five Arabidopsis chromosomes are depicted as lines in a pentagonal layout with roman numerals placed at the 0 cM position for each chromosome. Arrows to the outside of each chromosome show the positions of the identified QTLs. Inside each arrow, 1 indicates that the QTL was detected at 30 DPG, 2 for 35 DPG, 3 for 42 DPG and F for all DPG. Arrows for loci where the Bay-0 allele has a positive effect point away from the chromosomes while arrows for QTLs with negative allele substitution values point inward. The QTLs are named according to the nomenclature used in the text. Significant epistatic interactions are illustrated with arrows inside the pentagon connecting the interacting loci and numbered to indicate the DPG at which the interaction was detected. A) QTLs and epistasis affecting epithionitrile proportions. B) QTLs and epistasis affecting simple nitrile proportions.

To identify loci controlling the diversity of structural outcomes in Bay-0 and Sha, we independently mapped QTLs for all traits at 30, 35, and 42 DPG. Analysis of epithionitrile proportions for exogenous allyl glucosinolate revealed nine loci (Figure 3A, Table S3), including seven novel QTLs and the previously identified *ESP* and *ESM1* loci [22],[23]. Three loci (*ESP*, *GSL.Activ.II.13*, and *ESM1*) were detected in the RILs at all three DPG, although the additive effect of *ESM1* switched direction from promoting nitrile formation at 42 DPG relative to promoting isothiocyanate formation at 30 and 35 DPG. In spite of the fact that Bay-0 lacks functional *ESP*, four QTLs showed a positive impact of the Bay-0 allele on epithionitrile production, which is consistent with the observed transgressive segregation in the RILs (Figures 2D and 3A).

We identified ten loci affecting the proportion of simple nitrile structures produced from exogenous allyl glucosinolate, including *ESP*, *ESM1* and eight novel loci (Figure 3B, Table S3). The average allelic substitution effect of these QTLs was 27% and the median was 20%. *ESP*, *ESM1* and six of the novel loci overlapped with epithionitrile production QTLs with all but *ESP* showing the same direction of allelic effect upon epithionitrile and nitrile production (Figure 3). Six of the simple nitrile proportion QTLs were significant at a single DPG, three were detected at two consecutive DPG, and one locus (*GSL.Activ.II.13*) was detected in all three QTL maps. Increased simple nitrile proportions were fairly evenly distributed between the Bay-0 and Sha alleles (Figure 3B). One locus (*GSL.Activ.IV.16*) exhibited significant additive effects in opposite directions at 30 and 35 DPG (Figure 3B). Isothiocyanates identified a combination of the nitrile and epithionitrile QTLs (Figure S1). This suggests that the genetic architecture underlying glucosinolate activation is much more complex than the two locus model previously assumed [22],[41].

### Endogenous Glucosinolate Activation

We proceeded to compare QTLs identified using the exogenous allyl glucosinolate to those identified with the endogenous but-3-enyl and 4-methylsulfinylbutyl glucosinolate, the two glucosinolates with the highest level of accumulation in this population. The analysis of structural outcomes of endogenous glucosinolate activation is complicated by independent assortment at the *GSL.Elong* and *GSL.AOP* biosynthetic loci, limiting each measurable endogenous glucosinolate to one quarter of these RILs [38],[42],[43]. QTL analysis of endogenous but-3-enyl glucosinolate activation detected three loci and nine epistatic interactions affecting simple nitrile formation, and only *ESP* and three epistatic interactions for epithionitrile proportions. All QTLs were consistent with those observed using the exogenous allyl glucosinolate (Figure 3A and Table S3).

QTL analysis of 4-methylsulfinylbutyl glucosinolate activation identified three loci affecting simple nitrile formation, which were all identified using exogenous allyl glucosinolate (Table S3). The Sha allele of *ESP* increased simple nitrile formation from this glucosinolate, which lacks a terminal double bond and cannot form epithionitrile structures. In contrast, the non-functional Bay-0 allele increases simple nitrile formation from the exogenous allyl glucosinolate, possibly because this eliminates substrate competition between *AtNSP* and *ESP*. As such, the use of exogenous allyl glucosinolate provides the greatest power to independently map both structural outcomes utilizing the entire RIL population.

### QTL×DPG Interactions

To test how plant age in DPG altered QTL identification, we conducted an ANOVA analysis of the significant genetic loci using the data from all three assays in a single model. Each genotype is replicated within each DPG allowing for a test of marker×DPG interactions. Analysis of epithionitrile proportions using the full data set identified five marker×DPG interactions, suggesting that these loci may be involved in controlling the increase in epithionitrile formation observed from 30 to 42 DPG (Table S3). Analysis of simple nitrile proportions in the full data set also detected five significant QTL×DPG effects, suggesting age dependent regulation of simple nitrile rearrangements (Table S3). Three of the marker×DPG interactions significantly affected both simple nitrile and epithionitrile proportions, possibly as a consequence of these two rearrangements competing for the same pool of substrate or co-regulation of the two glucosinolate activation outcomes. These loci with DPG interactions provide the potential to begin understanding how ontogenic variation and genetic variation interact at the molecular level.

### Numerous Epistatic Interactions Influence Glucosinolate Activation

**Figure 4 pgen-1000234-g004:**
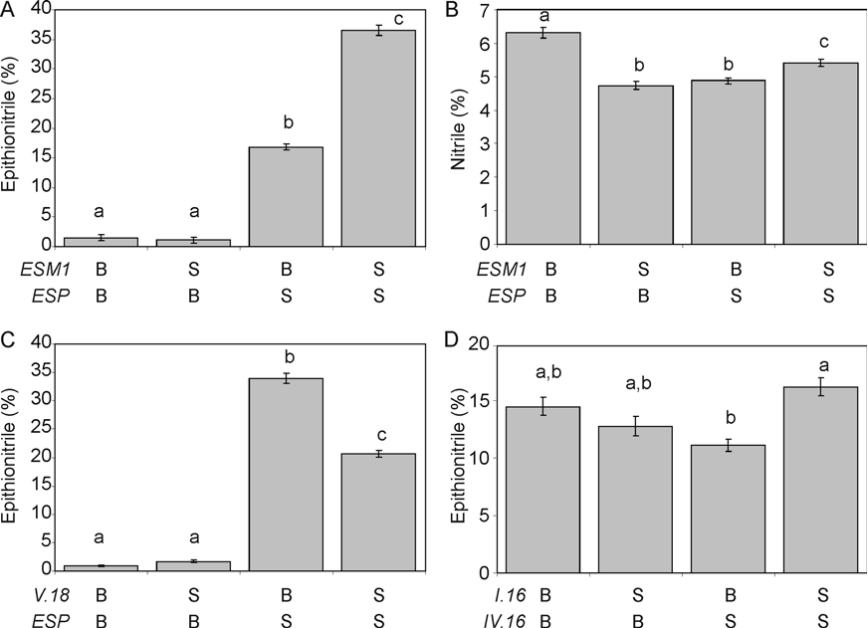
Phenotypic Consequences of Epistatic Interactions in the Structural Outcome of Glucosinolate Activation. We obtained the mean phenotypic values for four examples of epistatic interactions controlling the structural outcome of glucosinolate activation. The mean value for the listed structural outcome and standard error in each genotypic class are shown for each pair wise genotypic class in the displayed interaction. Different letters indicate statistically significant differences from the other genotypic class within an interaction. A) Interaction of *ESP×ESM1* for percent epithionitriles. B) Interaction of *ESP×ESM1* for percent simple nitriles. C) Interaction of *ESP×GSL.Activ.V.18* for percent epithionitriles. D) Interaction of *GSL.Activ.IV.16×GSL.Activ.I.16* for percent epithionitriles.

Given the numerous QTLs controlling glucosinolate activation and the requirement of a functional allele at the *ESP* locus for epithionitrile production, we hypothesized that there would be significant epistasis affecting structural outcomes in this mapping population. We utilized an ANOVA to test all possible pair wise QTL interactions for significant epistasis. We identified a total of eleven different pair wise epistatic interactions for epithionitrile proportion, including the previously described *ESP×ESM1* interaction [22],[23] (Figure 3A, Table S3). Consistent with the requirement of functional *ESP* for epithionitrile production, all other QTLs for epithionitrile formation showed a significant epistatic interaction with *ESP* for at least one of these data sets (Table S3). Epistatic interactions involving *ESP* represent classical epistasis, where genotypes with the nonfunctional Bay-0 *ESP* allele produce no detectable epithionitriles, hiding the function of the interacting loci (Figure 4A/C). This suggests that the QTLs epistatic to *ESP* may function to modulate the activity of the functional Sha allele of *ESP*. Interestingly, the highest levels of epithionitriles were not exclusively observed in RILs with Sha genotypes at the interacting locus. For example, the *ESP×GSL.Activ.V.18* interaction produced the highest epithionitrile proportions in lines with the Bay-0 allele at *GSL.Activ.V.18* (Figure 4C).

While all epithionitrile QTLs were epistatic to *ESP*, four epistatic interactions were detected for epithionitrile proportion that did not involve *ESP* (Figure 3B and 4D and Table S3). These interactions were examples of quantitative epistasis, where the effects of one locus was modified by the other locus in a quantitative manner, rather than the absolute dependence of one locus on the other locus as exhibited by interactions involving *ESP* (Figure 4D versus 4A/C). Additionally, these epistatic interactions formed networks such that *GSL.Activ.II.13*, *GSL.Activ.II.61* and *ESM1* showed all possible pair wise epistatic interactions with each other as well as with *ESP* (Figure 3B and Figure 4B). Likewise, a similar network involves *GSL.Activ.I.16×GSL.Activ.IV.16* and *ESP* (Figure 3). This suggests that complex epistasis may begin to identify underlying regulatory or protein interaction networks controlling the structural outcome of glucosinolate activation.

Simple nitrile proportion from exogenous allyl glucosinolate identified fewer epistatic interactions than epithionitrile formation with most interactions also involving the *ESP* locus. The *ESP×ESM1* epistatic interaction affected both simple nitrile and epithionitrile proportion, but with different effects on each structural proportion (Figure 4 A and B). The lower number of epistatic interactions for simple nitrile formation is partly explained by the lower variation present for simple nitrile formation within these RILs (Figure 2A/B).

### QTL Validation using HIFs

**Figure 5 pgen-1000234-g005:**
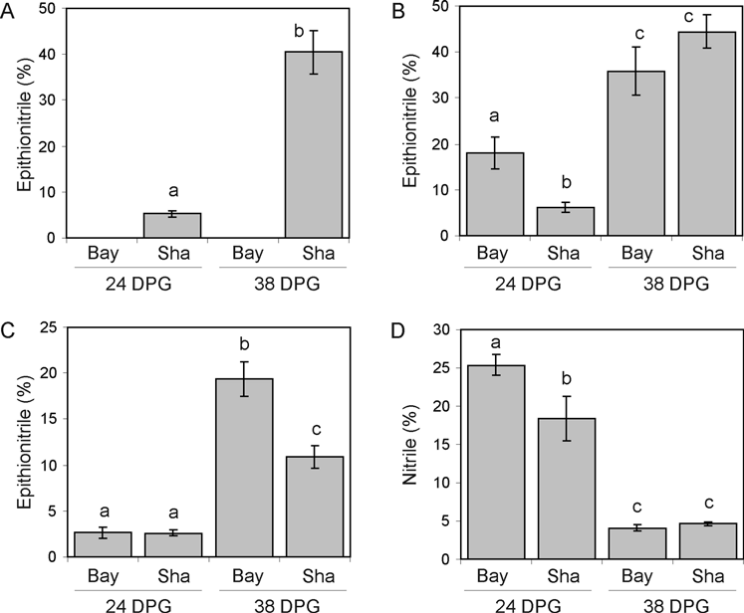
HIF Validation of Novel QTLs. The structural outcome of glucosinolate activation for allyl glucosinolate was assayed in HIFs to confirm the effect of specific QTLs, as well as QTL×DPG interactions. Average values are shown with standard errors (n = 6 per bar). Significant differences (*P*<0.05) are indicated by different letters. A) *ESP* controls percent epithionitriles in HIF425. B) *GSL.Activ.V.18* controls percent epithionitriles in HIF157. C) *GSL.Activ.V.48* controls percent epithionitriles in HIF350. D) *GSL.Activ.V.18* controls percent simple nitriles in HIF213.

To provide additional support for the identified *GSL.Activ* QTLs, we obtained HIFs that contain appropriate variation for seven of the loci detected in this study (Table S1). For the *GSL.Activ.II.13* and *GSL.Activ.III.64* loci, the available HIFs only contained a non-functional *ESP*, thus we were unable to test the effects of these loci on epithionitrile production. We assayed glucosinolate activation in each HIF line at 24 and 38 DPG, to confirm the QTLs and any interaction between the QTLs and DPG. The HIFs confirmed seven of the *GSL.Activ* QTLs. This included four loci for epithionitrile formation, two for simple nitrile production and several for the total production of nitriles or isothiocyanates (Figure 5, Table 2, Table S5). HIF-241 and HIF-425 vary for *ESP* and confirm that a functional Sha allele is necessary for epithionitrile production (Figure 5A, Table S5). Interestingly, the efficiency of epithionitrile formation significantly differs between these two HIFs (*P* = 0.047 for HIF×*ESP* genotype and *P* = 0.048 for HIF×*ESP* genotype×DPG), confirming the presence of background *ESP* modifiers. The level of validation observed in the HIF analysis is strongly supportive of the QTL mapping results, as each HIF genotype was only analyzed in six-fold replication per DPG whereas each marker genotype in the RIL study was analyzed in roughly 200-fold replication, lending more power to the RIL analysis.

**Table 2:** HIF Analysis of Structural Outcomes.

			Simple Nitrile		Epithionitrile		Isothiocyanate	
Locus	HIF	*ESP*	G	G×D	G	G×D	G	G×D
*GSL.Activ.I.16*	071	Sha	*- -*	*- -*	*- -*	*- -*	*- -*	*- -*
	194	Sha	*- -*	*a -*	*- P*	*- P*	*A p*	*a P*
*ESP*	241	Het	*a -*	*a -*	*A P*	*A P*	*A P*	*A P*
	425	Het	*a -*	*- -*	*A P*	*A P*	*A P*	*A P*
*GSL.Activ.II.13*	364	Bay	*a p*	*nd*	*	*	*a p*	*nd*
*GSL.Activ.II.42*	163	Sha	*- -*	*- -*	*- -*	*- -*	*- -*	*- -*
*ESM1*	338	Sha	*- -*	*nd*	*- -*	*nd*	*- -*	*nd*
*GSL.Activ.III.64*	244	Bay	*a -*	*nd*	*	*	*- -*	*nd*
*GSL.Activ.IV.55*	077	Sha	*- -*	*- -*	*- -*	*- -*	*a -*	*A -*
	191	Sha	*- -*	*nd*	*- -*	*nd*	*- -*	*nd*
*GSL.Activ.V.18*	213	Bay	*- -*	*- p*	*	*	*- -*	*- p*
	157	Sha	*- -*	*- -*	*- -*	*a P*	*- -*	*- p*
	340	Sha	*- -*	*- -*	*- -*	*- -*	*- -*	*- -*
*GSL.Activ.V.48*	350	Sha	*- -*	*- -*	*A P*	*A P*	*- -*	*- -*

Locus indicates the QTL region that varies in each HIF (Table S1 and S5). HIF gives the HIF number and *ESP* indicates the allelic status at the *ESP* QTL in each HIF. A significant effect of genotype (G) and genotype×DPG (G×D) on the absolute quantity of each structure is indicated with *a* or *A* for *P*-values below 0.05 and 0.01 respectively, and significant effects on the proportion of each structure is indicated with *p* or *P*. Dashes indicate non-significant effects. (*nd*) indicates HIFs for which it was not possible to assess G×D, as they were measured at 38 dpg only. (^*^) indicates no epithionitrile formation is possible in HIFs lacking functional *ESP*.

The original RIL QTL analysis did not replicate each line within each DPG, and as such, we designed the HIF analysis to confirm that these QTLs do interact with the plant age in DPG. All four confirmed epithionitrile proportion QTLs exhibited significant genotype×DPG effects (Figure 5, Table 2 and Table S5). For example, HIF-157 which varies for *GSL.Activ.V.18*, showed a significant difference in epithionitrile proportions between alleles at 24 but not 38 DPG (Figure 5B and Table S5). In contrast, the locus *GSL.Activ.V.48* in HIF-350 showed no difference in epithionitrile proportions between alleles at 24 DPG, but a significant effect at 38 DPG in agreement with the QTL prediction from the RIL analysis (Figures 3A, 5C and Table S5). These results confirm that we have identified ontogenic dependent QTLs in our study.

### HIF Analysis Reveals an Additional QTL Tightly Linked to *ESP*

**Figure 6:**
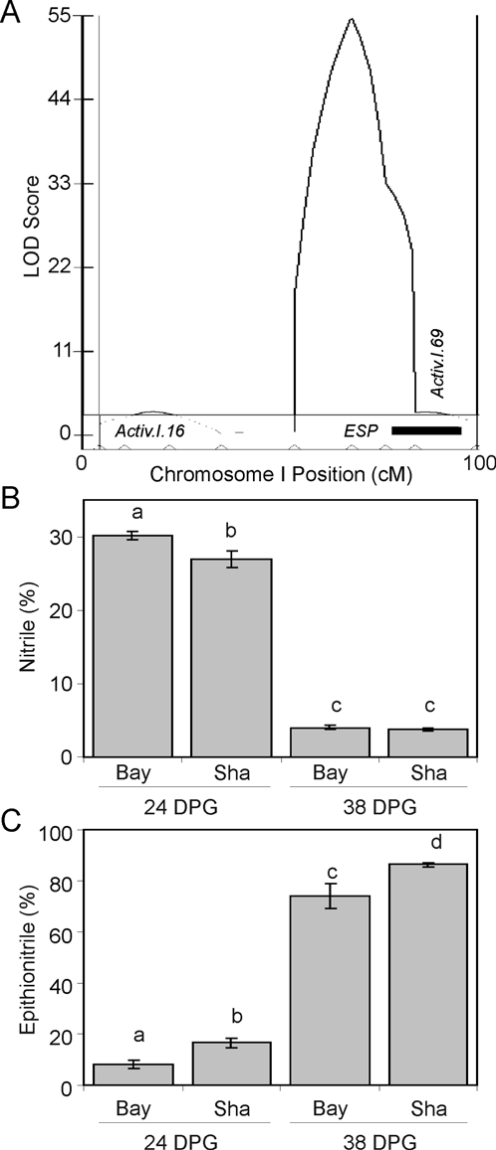
HIF Analysis Identifies an Additional Locus, *GSL.Activ.I.69*. HIF149 was utilized to test a putative QTL near the *ESP* locus. Glucosinolate activation was assayed using allyl glucosinolate and the mean and standard error are shown (N = 6). Significant differences (*P*<0.05) are indicated by different letters. A) Illustration of the described shoulder in the LOD profile near the *ESP* locus on chromosome I generated by CIM based QTLcartographer analysis (35 DPG is illustrated). The black bar shows the region of the shoulder that varies in HIF149. B) Effect of HIF149 upon simple nitrile production. C) Effect of HIF149 upon epithionitrile production.

QTL mapping analysis of epithionitrile proportion consistently produced a large peak in the LOD plot at the *ESP* locus but there was also frequently a small shoulder (Figure 6A). This could be explained by residual significance from the large-effect *ESP* locus or suggest the presence of a tightly linked QTL in this region. Due to tight genetic linkage with *ESP*, we did not include this putative locus in our statistical models. We did however identify two HIFs in this region, HIF149 with functional *ESP* and HIF107 with non-functional *ESP*. These HIFs allow us to test for the existence of this additional locus as well as its potential dependency upon *ESP* (Table S1). Analysis of structural outcomes in HIF149 confirmed the existence of an additional QTL teleomeric of *ESP* on Arabidopsis chromosome I. This QTL, *GSL.Activ.I.69*, affects both simple nitrile and epithionitrile proportions (Table S5 and Figure 6B/C). *GSL.Activ.I.69* also displays an age dependent effect on simple nitrile proportion, but not epithionitrile proportion (Table S5 and Figure 6B/C). Interestingly, HIF107 did not identify a significant QTL, suggesting that *GSL.Activ.I.69* is epistatic to *ESP* (Table S5). This HIF analysis of a QTL shoulder suggests there may be even further additional QTLs for glucosinolate activation in this RIL population.

### eQTLs for Glucosinolate Activation Genes

The *TGG1* and *TGG2* myrosinases, *ESP*, and *ESM1* are the primary genes with a demonstrated role in controlling glucosinolate activation and associated structural outcomes within the Arabidopsis rosette [22]–[25],[27],[30],[31]. However, these genes and the *MBPs* form gene families in Arabidopsis and some of these uncharacterized homologs may determine the genetic variation observed in the structural outcome of glucosinolate activation. To identify any potential expression level polymorphisms in these four gene families that may control the glucosinolate activation QTLs, we identified eQTLs for the full list of potential glucosinolate activation homologs (Table S2) [37].

We first obtained the estimated heritability for transcripts encoding these potential glucosinolate activation genes [37]. The average transcript heritability for the 30 measurable probe sets was 63.0%, which is significantly higher than the genome wide average transcript heritability (t-test, *P* = 0.045). Further, the glucosinolate activation genes had more eQTLs, both *cis* and *trans* than the average Arabidopsis transcript. This excess was most dramatic with *trans*-acting eQTLs, with an average of 3.1 *trans*-eQTLs detected for each glucosinolate activation gene in comparison to the average Arabidopsis transcript, with 1.5 *trans*-eQTLs in this population [37]. These results agree with previous studies showing the transcripts for glucosinolate biosynthetic genes had higher heritability and variance than the average Arabidopsis transcript [38],[44].

**Figure 7:**
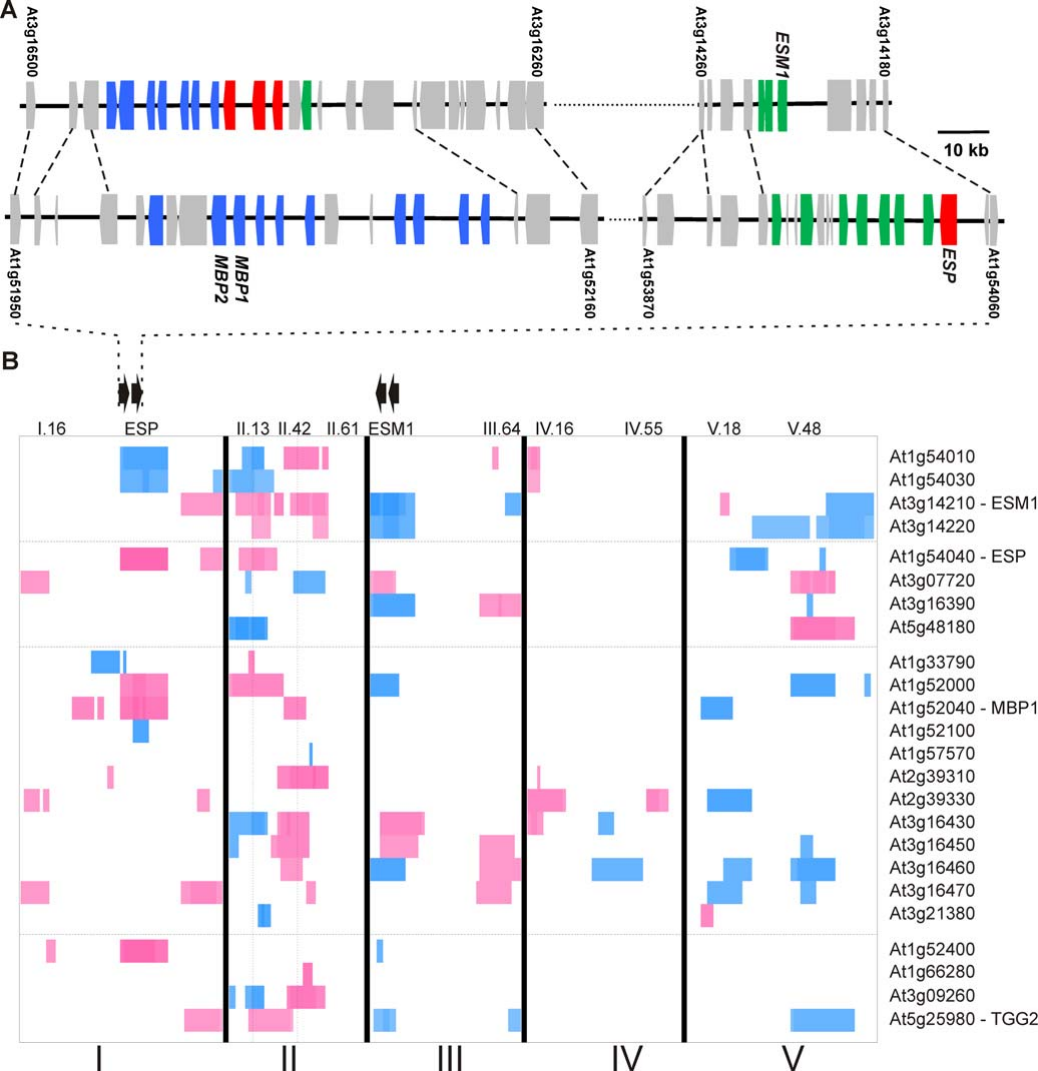
Genomic Duplication and eQTLs for Glucosinolate Activation Genes. Phylogenetic analysis was used to identify homologs of the four known families of glucosinolate activation genes (Myrosinase, *ESP*, *ESM1* and *MBPs*) within Arabidopsis. The genomic position of these genes and their associated eQTLs within this population were then identified and are plotted below. A) The genomic arrangement of the glucosinolate activation genes at the identified genomic duplication underlying the *ESM1* (top) and *ESP* (bottom) QTLs. Genes are colored based on the glucosinolate activation gene with which they share homology; *ESP* (red), *ESM1* (green), and *MBP* (blue). Genomic relationships for eight additional paralogous sequences are indicated by dashed lines. The AGI locus designation for the genes used as the beginning and end of each illustrated regions are indicated, and the loci corresponding to *ESP*, *ESM1*, *MBP1* and *2* are labeled, with the scale indicated. B) eQTLs for the identified Myrosinase, *ESM1*, *ESP*, and *MBP* homologs are shown [37]. The five Arabidopsis chromosomes are indicated with roman numerals and represented contiguously from left to right. The position of the identified structural outcome QTLs are indicated at the top of the heat plots. Horizontal lines separate the four gene families with the defining member labeled. Within each family the genes are ordered by physical position from top to bottom. eQTLs shaded blue indicate that increased transcript accumulation for that gene is associated with the Bay-0 allele, and red indicates Sha, with the intensity of the color proportional to the magnitude of the additive effects. The double black arrows show the orientation and position of the tandem gene clusters at the *ESP* and *ESM1* loci. The vertical dashed lines show the likely position for the *GSL.Activ.II.13* and *42* loci.

The previous analysis of eQTLs within the Bay-0×Sha RIL population was conducted at 35 DPG in the same growth chamber allowing their direct comparison [37]. The eQTLs controlling transcript accumulation of the glucosinolate activation genes revealed eQTL clusters collocating with ten of the eleven structural proportion QTLs (Figure 7B). The eQTL clusters partitioned into both *cis* and *trans*-eQTL clusters. The two *cis*-eQTL clusters are associated with the genomic regions around *ESP* and *ESM1*, which contain several *ESP*, *ESM1*, and *MBP* homologues but do not overlap with known trans-eQTL hotspots (Figure 7A and B [see the black arrows])[37]. These regions appear to be the result of two separate whole-genome duplications that copied an original region containing the ancestral genes for *ESP*, *ESM1* and the *MBPs* (Figure 7A). These consecutive duplications generated four genomic regions that contain additional non-glucosinolate genes whose paralogs also have a conserved order (Figure 7A). In these two *cis*-eQTL clusters, we found six and eight *cis*-eQTLs for putative glucosinolate activation genes in the *ESP* and *ESM1* regions respectively (Figure 7). While variation in *ESP* and *ESM1* have been shown to control glucosinolate activation phenotypes, these local *cis*-eQTL clusters suggest that additional genes at each locus could contribute to the effects on the structural outcome of glucosinolate activation.

In addition to *cis*-eQTL clusters, there were several *GSL.Activ* loci that co-located with clusters of *trans*-eQTLs suggesting that these may be regulatory loci with a measurable effect on gene expression and structural outcomes. In particular, *GSL.Activ.II.13* collocated with a large *trans*-eQTL cluster but not with the genomic position of any of the identified glucosinolate activation gene homologs (Figure 7B – double black arrows highlight homologue clusters). Interestingly, this locus also collocates with a large trans-eQTL hotspot controls ∼1,200 genes suggesting that it may contain a polymorphism in a pleiotropic developmental regulator [37]. Genetic variation at the *GSL.Activ.II.13* locus alters the expression of all four *ESM1* homologues, three of four *ESP* homologues, four *MBP*s, and two myrosinases. A closely linked locus, *GSL.Activ.II.42*, also collocates with a large *trans*-eQTL cluster controlling two myrosinases, eight of twenty potentially interacting genes and ∼2,500 other genes (Figure 7B)[37]. Thus, these two loci show that global regulatory loci can have a measurable consequence on glucosinolate activation. Interestingly, the *GSL.Activ.I.69* locus identified in the HIF analysis is associated with eQTLs controlling transcript accumulation for *TGG2*, *ESP*, *ESM1* and an *MBP* (*At3g16470*), suggesting that the *GSL.Activ.I.69* may also be a regulatory locus. However, it does not co-locate with a previously identified global *trans*-eQTL hotspot suggesting that it may be more specific to glucosinolate activation [37].

## Discussion

The structural outcome of glucosinolate activation strongly influences plant defense, and had been thought to be predominantly controlled by genetic variation in *ESP* and its epistatic modifier locus, *ESM1* [22], [23], [28]–[30]. Recently this model has been shown to be overly simplistic by the identification of interactions between developmental, environmental and genetic factors influencing the regulation of the structural outcomes in Arabidopsis, Brassica and Nasturtium [30], [32], [45]–[48]. These analyses suggested that the mixture of biologically active product structures is generated by two biochemically distinct rearrangements that divert the glucosinolate substrate away from the default isothiocyanate structure, each subject to complex regulatory patterns in rosette leaves. As such, we conducted QTL mapping analysis to simultaneously map loci controlling both simple nitrile and epithionitrile formation using allyl glucosinolate in the Bay-0×Sha RIL population. The power afforded by this population containing a moderate number of independent lines revealed considerable additional complexity underlying the critical rearrangement step in glucosinolate activation.

Our analysis identified twelve loci and 17 epistatic interactions controlling the mixture of activation structures produced from allyl glucosinolate, including the previously described *ESP* and *ESM1* QTLs (Figures 3 to [Fig pgen-1000234-g004] [Fig pgen-1000234-g005] 6, Table 2, Table S3 and S5). HIF analysis confirmed seven loci as well as their proposed interaction with plant development (Figure 5 and 6, Table 2 and S5). We also found extensive collocation between eQTLs for the genes potentially involved in glucosinolate activation and the structural outcome phenotypic QTLs (Figure 7 and Table S2). These results support a model wherein control of structural outcomes during glucosinolate activation results from multi-locus regulation of both simple nitrile and epithionitrile formation and the interplay between these competing rearrangement processes generates the final mixture of biologically active structures.

### Age Dependent Regulation of the Structural Outcome of Glucosinolate Activation

The Bay-0 and Sha parents had previous been shown to have different ontogenic control of glucosinolate activation [32]. Our analysis of epithionitrile proportions revealed several differences between 30, 35 and 42 DPG, and analysis of the full data set identified seven significant QTL×DPG interactions (Figure 3A and Table S3). Further, direct assessment of glucosinolate activation at two DPG in the HIFs confirmed these QTL×DPG interactions (Figure 5, Table 2, S3 and S5). This suggests that these loci are responsible for the age dependent regulation of epithionitrile production, and may act to regulate *ESP* expression. Similar levels of ontogenic QTL dependency were identified for simple nitrile production in spite of the absence of ontogenic variation between Bay-0 and Sha for simple nitrile production (Figures 2A/B and 3B and Table S3). Thus, transgressive segregation can also occur for interaction terms in QTL analysis.

### Epistasis and *trans*-eQTLs

Extensive epistatic interactions controlling glucosinolate structural outcomes were detected, many involving *ESP* that may represent classical epistasis where the phenotypic effects of the second locus on epithionitrile production are fully masked in the absence of functional *ESP* (Figures 3A and 4A/C, and Table S3). These interactions formed networks wherein all possible pair wise interactions were detected (Figure 3A). One such epistatic network involved the known structural genes *ESP* and *ESM1*, with *GSL.Activ.II.13* and *GSL.Activ.II.42*. Interestingly, *GSL.Activ.II.13* and *GSL.Activ.II.42* appear to be *trans* regulatory loci that control the expression of *ESP* and *ESM1*, several other putative glucosinolate activation genes and thousands of other genes suggesting (Figure 7B)[37]. This suggests that this epistatic network is a combination of variation in two master regulatory loci, as well as variation in the genes that they regulate, *ESP* and *ESM1*. Further evidence for *trans* regulation underlying epistasis comes from the *ESP*×*GSL.Activ.V.18* interaction, where *GSL.Activ.V.18* collocates with an *ESP trans*-eQTL (Figure 4C and 7B). The connection of regulators and their regulated genes in a quantitative epistatic network suggests that it may be possible to use quantitative epistasis in a manner similar to Mendelian epistasis to begin defining molecular networks and their influence upon the final phenotype. Further work will be required to validate if these regulators are directly or indirectly affecting transcript accumulation of *ESP*, *ESM1* and the other homologs.

One complication that occurs from the observed level of epistasis is a diminished statistical power to identify loci. As such, the 400 lines used may underestimate the true extent of epistasis for the structural outcome of glucosinolate activation. As a consequence of this extensive epistasis, we hesitate to eliminate QTLs not confirmed by HIF analysis as candidate loci. The genetic background of each HIF consists of a random mixture of fixed Bay-0 and Sha genotypes at all regions outside of the focal locus, and it is therefore likely that some of the available HIFs have unfavorable genotypes at interacting loci. Of the four QTLs with multiple HIFs available, three were confirmed in some backgrounds and not in others, supporting a genetic background effect (Table 2). In particular, analysis of simple nitrile proportion appears complicated by *ESP* genotype. Both HIFs with significant effects on simple nitrile proportions lacked *ESP* activity, suggesting that the ability to detect small effects on simple nitrile formation can be negatively impacted as a consequence of reduced flux in the presence of the competing epithionitrile rearrangement.

### Structural Outcome QTLs May Result from an Ancient Duplication and Subsequent Neo-Functionalization

Analysis of the genomic regions underlying *ESP* and *ESM1* revealed two distinct and tightly linked clusters of structural specifiers and myrosinase interacting proteins at each locus (Figure 7A). These linked clusters appear to be the product of an ancestral locus, which contained *ESP*, *ESM1*, and an *MBP* and underwent a tandem duplication followed by segmental duplication to generate the *ESP* and *ESM1* loci, with the subsequent loss of some paralogs. These four genomic regions contain the majority of the *ESP*, *ESM1* and *MBP* homologs but differ in their specific composition. Further, a number of *cis*-eQTL were detected for these genes, and the associated QTLs have divergent effects on the structural outcome of glucosinolate activation [22],[23]. The presence of a large number of duplicated homologs suggests that these QTL may be complex loci with numerous tightly linked polymorphisms contributing to the observed phenotypic effects. Support for this idea comes from the observation that while the *ESP* and *ESM1* proteins were shown to explain most of the effects of their respective QTLs, complementation of both QTLs did not completely recapitulate the phenotypes associated with each QTL [23],[42].

The association of genomic duplications with glucosinolate activation QTLs suggests that such duplications may facilitate quantitative genetic variation by creating duplicate genes. The duplicate genes can then undergo genetic sub-functionalization such that the genes have differential functions across natural genotypes [44], [49]–[51]. This role of genome duplications and QTL association has been previously seen in polyploid plants but not characterized in diploids [52],[53]. This relationship between genome duplications and QTLs requires the analysis of more traits and cloning of more loci to establish the generality of this connection.

### Simple Nitrile and Epithionitrile Rearrangements Involve Distinct Chemistry but Overlapping Genetics

Nine of twelve QTLs affected both simple nitrile and epithionitrile proportions formed from allyl glucosinolate (Figure 3 and 6, and Table S3). Additionally, four epistatic interactions were detected in both the simple nitrile and epithionitrile structural outcome data (Figure 3 and 4 and Table S3). While there was considerable overlap in the QTLs for the two distinct structural outcomes, there was not complete correspondence in the directionality of their effects. The majority of the QTLs altered the accumulation of nitrile and epithionitrile in the same direction, but the *ESP* locus had opposite effects on simple nitrile and epithionitrile proportions (Figure 3). In those cases where the same pair wise interactions via the ESP locus affected both simple nitrile and epithionitrile formation, they affected each proportion differently, supporting the concept of two independent rearrangements competing for the same substrate. This suggests that simple nitrile and epithionitrile formation share regulatory loci, but that there are likely separate proteins producing nitrile and epithionitrile structures from allyl glucosinolate. This is in agreement with previous observations suggesting the presence of an unidentified simple nitrile forming enzyme in Arabidopsis [23],[25],[32].

### Conclusion

This study shows that there is considerable natural genetic variation controlling the age dependent regulation of structural outcomes in Arabidopsis [32]. Elucidating the basis of this regulation is necessary to obtain a better understanding of the evolution and ecological significance of developmental trajectories in this important plant defense system. Further, we describe epistatic networks that appear to link regulatory loci with the genes that they regulate. Future analyses will be required to test if quantitative epistasis can be used to generate networks in a fashion similar to Mendelian epistasis but this has potential applications in most species. Finally, the potential for whole genome duplications to be associated with multiple QTLs for the same trait may help to enhance the rate at which additional QTL can be cloned. Once one QTL is cloned for a given trait, it may immediately suggest candidate genes for QTL in genomic regions that share an ancestry through whole genome duplications. The glucosinolate system is a useful model system for quantitative genetics to begin addressing these fundamental issues in quantitative genetics, ecology and evolution.

## Materials and Methods

### Mapping Population

The population of 411 Bay-0×Sha RILs [33] were chosen for QTL mapping analysis of the structural outcome of glucosinolate activation. The parents of this population differ in their glucosinolate profile and content, as well as in the structures formed upon glucosinolate activation and the developmental regulation of the structural outcome following glucosinolate activation [32],[38]. A subset of 212 lines from this population have also been analyzed for variation in gene expression [37], enabling comparison of gene expression to phenotypic variation. Finally, there are available HIFs, pairs of near isogenic lines fixed for alternate alleles at a single locus in an otherwise identical recombinant inbred background, which offer the opportunity to validate some of the QTLs detected in this study [54].

### RIL Growth Conditions and Experimental Design

Seeds were imbibed and cold stratified at 4 degrees for three days to break dormancy. All seeds were sown directly onto Premier ProMix B potting soil (Premier Brands, Inc., Red Hill, Pennsylvania) in 36-cell (approximately 125 cm^3^ soil per cell) flats, and grown in controlled environment chambers at 20°C with 8 h light at 100–120 µEi. Each flat contained one Bay-0 and Sha parents. All plants were free of insect pests by visual inspection. The population was grown three independent times to independently phenotype the structural outcome of glucosinolate activation at 30, 35, and 42 days DPG. These DPG were chosen because day 30 represents the attainment of Stage 1.10 or 10 mature leaves per plant for both the parents and all RILs. Further, day 42 is one week away from the earliest RIL flowering (Stage 5.10) in our environmental conditions [32],[34],[35]. Thus, this range of DPG allows us to focus on developmental changes in what is typically considered a static rosette rather than query larger ontogenic shifts such as leaving logarithmic growth or the flowering transition. Within a two hour time frame centering on dawn, the rosette leaves from each and every RIL at each DPG were harvested and phenotyped for the structural outcome of glucosinolate activation. Previous work had shown that glucosinolate activation is regulated by rosette age and not the age of individual leaves within a rosette [32] .

### Analysis of Glucosinolate Activation Product Structures

The structural outcome of glucosinolate activation was assayed using a modified version of the previously published protocol [22],[23]. Briefly, the three fully expanded rosette leaves from a single plant were harvested and crushed in an 8 mL reaction vial containing 1 mL of 100 mM MES buffer at pH 6.0 and 0.4 µmol of allyl glucosinolate. The three leaves were consistently the first, fourth and seventh fully expanded leaf to provide a sampling of different ages. Previous work had shown that these three leaf ages had similar glucosinolate activation that was determined by the rosette age and not the leaf age [32]. This allows us to focus on rosette age rather than leaf age although the two may be intricately linked in some fashion. Exogenous allyl glucosinolate was added to enable comparisons of structural outcomes using a common substrate for all RILs despite the segregating biosynthetic variation [38]. Further, the allyl glucosinolate allows us to measure all three potential glucosinolate activation endpoints, isothiocyanate, epithionitrile or simple nitrile, whereas half the RILs do not have this capacity due to the lack of alkenyl glucosinolates [32],[38]. Upon complete tissue homogenization the reaction vial was capped and incubated for five minutes. The reaction was stopped and glucosinolate activation products extracted with 4 mL of dichloromethane. The organic phase was removed, dried and concentrated to 200 µL for gas chromatography (GC) analysis using an Agilent HP 5890 with a flame ionization detector [22].

Peak identities were confirmed using a GC-mass spectral detector (Agilent HP 6890 with an Agilent 5973N MSD), by comparison with published mass spectra [55]. Quantification was conducted using published response factors that were corrected using propyl isothiocyanate standards as previously described [22]. Structural outcomes are reported as the percent of simple nitrile, epithionitrile, or isothiocyanate products for a particular glucosinolate. For instance, the percent simple nitrile for allyl glucosinolate is defined as [allyl simple nitrile] / [allyl simple nitrile+allyl epithionitrile+allyl isothiocyanate]. Proper chemical names for this equation are allyl simple nitrile is 3-butenyl nitrile and allyl epithionitrile is 2,4-epithiobutyl nitrile. Each structural outcome for each glucosinolate was measured using a similar equation. By dividing the absolute amount of a particular structure by the sum of all possible products, the effects of myrosinase activity and differences in biosynthesis and accumulation of the endogenous substrates are cancelled, since they affect both the numerator and denominator equally. This assay is not a quantitative measure of total myrosinase activity because it reaches saturation for some samples.

### QTL Analysis

We obtained genotypes and genetic map information for the Bay-0×Sha RIL population from the Arabidopsis Biological Resource Center (ABRC; www.arabidopsis.org) [33]. To maximize our ability to detect QTLs, we utilized the data from each DPG experiment separately and as a combined data set. For each RIL, the proportion of each activation structure for each glucosinolate were independently used for QTL mapping within Windows QTL Cartographer v2.5 [56]–[58]. Although the proportions of glucosinolate activation structures obtained from a given substrate are not mathematically independent of one another, the simple nitrile and epithionitrile rearrangements can be separately measured for allyl glucosinolate, allowing simultaneous assessment of these partially independent processes [32]. Composite interval mapping (CIM) was implemented using Zmap (Model 6) with a 10 cM window and an interval mapping increment of 2 cM. The declaration of statistically significant QTL is based on permutation derived empirical thresholds using 1,000 permutations for each trait mapped [59],[60]. The Eqtl module of QTL Cartographer was used to automatically identify the location of each significant QTL for each trait [58].

To further test each QTL identified and query for potential epistasis, we conducted an ANOVA for the proportion of each glucosinolate activation structure. The markers most closely linked to each significant main-effect QTL were used as main effect cofactors. An automated SAS script then tested all main effects and all possible pair wise interactions between main-effect loci. Significance values were corrected for multiple testing within a model using false discovery rate adjustment within the automated script. The script returned all significance values as well as QTL main-effect estimates in terms of allelic substitution values (Table S3). In addition, the combined data were used to estimate the heritability of the different structural outcomes of glucosinolate activation. This was conducted using the general linear model procedure within SAS where broad sense heritability was defined as σ_g_/σ_p_ (Table 1), where σ_g_ is the estimated genetic variance for the structural proportion phenotypes among different genotypes in these RILs, and σ_p_ is the estimated phenotypic variance [3].

### Analysis of HIFs for QTL Validation

To confirm the identified QTLs in this study we obtained sixteen HIFs, corresponding to nine of the loci detected in this experiment, from INRA (http://dbsgap.versailles.inra.fr/portail) (Table S1). There were no HIFs available to test *GSL.Activ.IV.16* and *GSL.Activ.II.61*. Within each HIF, only the genotypes in the region of one QTL differ while the rest of the genome is a random homozygous mixture of Bay-0 and Sha genotypes. HIFs with the functional Sha allele at *ESP* can be used to test QTLs controlling both epithionitrile proportion and simple nitrile proportion. For most QTLs there was a HIF available with functional *ESP*, except for *GSL.Activ.II.13* and *GSL.Activ.III.64*. To test simple nitrile proportion QTLs for dependence on *ESP* genotype, separate HIFs with the functional Sha and non-functional Bay-0 alleles of *ESP* were chosen when possible.

Each HIF was planted with twelve independent biological replicates per allele per HIF. These were planted and grown under identical conditions as described above for the RIL population. For each HIF, six replicates of each genotype were assayed for structural outcomes at 24 DPG and six were assayed at 38 DPG to validate each QTL and survey for age dependence. These time points were chosen such that there was a sufficient developmental time difference to detect genotype×DPG effects but before epithionitrile formation reached saturation at the later time points. Due to poor germination, HIF191, 244, 338, and 364 were only assayed at 38 DPG.

The data for each class of glucosinolate activation product were analyzed for the effects of genotype, DPG, and DPG×genotype within each HIF using the general linear model procedure in SAS. Given that each HIF is a separate and independent test, we did not correct for multiple testing within these models. We also directly compared HIF241 to HIF425, two independent HIFs differing at the *ESP* QTL, to assess background dependent effects upon *ESP*.

### Analysis of Gene Expression QTLs

We used previously published sequence data to identify the known myrosinases and structural specifier genes [22]–[24],[61]. We utilized protein sequence data to identify Arabidopsis homologs of each major glucosinolate activation gene, myrosinase, *ESP*, *ESM1*, and *Myrosinase Binding Protein 1* (*MBP1*) and *MBP2*. For all four gene families we had two criteria to define a gene as potentially associated with glucosinolate activation. First, each included gene had to be similar to known genes based on a BLASTP score of at least e^−45^. Secondly, we further restricted this list to genes that were phylogenetically limited to Arabidopsis when protein sequences from the poplar, grape and rice plant genomes were included [62],[63]. This assumes that genes with more similar, non-cruciferous homologues are unlikely to be involved in glucosinolate activation as this system is not present in poplar, grape or rice (Table S2). Heritability, eQTL position, eQTL effect and transcript accumulation values were obtained from a previously published analysis of the Bay-0×Sha population [37]. Because these global transcription studies were conducted in the same mapping population grown under the same conditions and in the same growth chambers, it was possible to directly compare the gene expression and structural outcome data.

### Accession Numbers

There are no new accession numbers associated with this dataset. The microarray data set used in this study has been deposited at EBI ArrayExpress (http://www.ebi.ac.uk/arrayexpress/) under numbers E-TABM-126 and E-TABM-224.

## Supporting Information

Figure S1 QTLs Controlling the Isothiocyanate Outcome of Glucosinolate Activation. The five Arabidopsis chromosomes are depicted as lines in a pentagonal layout with roman numerals placed at the 0 cM position for each chromosome. Arrows to the outside of each chromosome show the position of the identified QTLs affecting isothiocyanate production. Inside each arrow, a number 1 indicates that the QTL was detected at 30 DPG, 2 for 35 DPG, and 3 for 42 DPG. Arrows for loci with positive allele substitution values for Bay-0 point away from the chromosomes while arrows for QTL with negative allele substitution values point inward. The QTLs are named according to the nomenclature used in the text. Significant epistatic interactions are illustrated with arrows inside the pentagon connecting the interacting loci and numbered to indicate the DPG at which the interaction was detected.
(2.90 MB TIF)

Table S1 HIF Genotypes. The genotype for each HIF line at the listed markers is provided. Heterozygous scores are in bold.
(0.03 MB XLS)

Table S2 Known and Putative Glucosinolate Activation Genes. Shown are the identified candidate genes for the different glucosinolate activation classes that contain a probeset on the Affymetrix ATH1 microarray.
(0.03 MB XLS)

Table S3 QTL for Glucosinolate Activation Traits. Main effect and DPG interaction provide the P value for the given QTL in relation to the listed trait. For the Epistasis analysis, 1 = a significant epistatic interaction at DPG30, 2 is for significance at DPG35, 3 is for significance at DPG42 and F is significant epistasis across all times. Allele substitution provides the estimated main effect of each QTL on the phenotype.
(0.04 MB XLS)

Table S4 Glucosinolate Activation with Allyl and Butenyl glucosinolates in the RILs. Phenotypic values for glucosinolate activation in the given RIL at the given day-post-germination (DPG).
(0.75 MB XLS)

Table S5 Summary of HIF Results. Shown are all statistical analyses on the HIFs with grey horizontal bars separating the different QTLs. P values for each term in the model as well as mean glucosinolate activation values and standard deviation are provided for each HIF.
(0.05 MB XLS)

## References

[1] Lynch M, Walsh B (1998). Genetics and analysis of quantitative traits.

[2] Mackay TFC (2001). The genetic architecture of quantitative traits.. Annu Rev Genet.

[3] Falconer DS, Mackay TFC (1996). Introduction to Quantitative Genetics.

[4] Kearsey MJ, Farquhar AGL (1998). QTL analysis in plants; where are we now?. Heredity.

[5] Holland JB (2007). Genetic architecture of complex traits in plants.. Curr Opin Plant Biol.

[6] Melchinger AE, Utz HF, Schon CC (1998). Quantitative trait locus (QTL) mapping using different testers and independent population samples in maize reveals low power of QTL detection and large bias in estimates of QTL effects.. Genetics.

[7] Stich B, Yu JM, Melchinger AE, Piepho HP, Utz HF (2007). Power to detect higher-order epistatic interactions in a metabolic pathway using a new mapping strategy.. Genetics.

[8] Visker M, Keizer LCP, Van Eck HJ, Jacobsen E, Colon LT (2003). Can the QTL for late blight resistance on potato chromosome 5 be attributed to foliage maturity type?. Theor Appl Genet.

[9] Clarke JD, Aarts N, Feys BJ, Dong XN, Parker JE (2001). Constitutive disease resistance requires EDS1 in the Arabidopsis mutants cpr1 and cpr6 and is partially EDS1-dependent in cpr5.. Plant J.

[10] Zhao JW, Meng JL (2003). Genetic analysis of loci associated with partial resistance to *Sclerotinia sclerotiorum* in rapeseed (*Brassica napus* L.).. Theor Appl Genet.

[11] Ahmadi N, Albar L, Pressoir G, Pinel A, Fargette D (2001). Genetic basis and mapping of the resistance to Rice yellow mottle virus. III. Analysis of QTL efficiency in introgressed progenies confirmed the hypothesis of complementary epistasis between two resistance QTLs.. Theor Appl Genet.

[12] Calenge F, Drouet D, Denance C, Van de Weg WE, Brisset MN (2005). Identification of a major QTL together with several minor additive or epistatic QTLs for resistance to fire blight in apple in two related progenies.. Theor Appl Genet.

[13] Coaker GL, Francis DM (2004). Mapping, genetic effects, and epistatic interaction of two bacterial canker resistance QTLs from *Lycopersicon hirsutum*.. Theor Appl Genet.

[14] Thabuis A, Palloix A, Servin B, Daubeze AM, Signoret P (2004). Marker-assisted introgression of 4 *Phytophthora capsici* resistance QTL alleles into a bell pepper line: validation of additive and epistatic effects.. Mol Breed.

[15] Thabuis A, Lefebvre V, Bernard G, Daubeze AM, Phaly T (2004). Phenotypic and molecular evaluation of a recurrent selection program for a polygenic resistance to *Phytophthora capsici* in pepper.. Theor Appl Genet.

[16] Martin GB, Brommonschenkel SH, Chunwongse J, Frary A, Ganal MW (1993). Map-based cloning of a protein-kinase gene conferring disease resistance in tomato.. Science.

[17] Malmberg RL, Mauricio R (2005). QTL-based evidence for the role of epistasis in evolution.. Genet Res.

[18] Malmberg RL, Held S, Waits A, Mauricio R (2005). Epistasis for fitness-related quantitative traits in *Arabidopsis thaliana* grown in the field and in the greenhouse.. Genetics.

[19] Fahey JW, Zalcmann AT, Talalay P (2001). The chemical diversity and distribution of glucosinolates and isothiocyanates among plants.. Phytochemistry.

[20] Louda S, Mole S, Rosenthal GA, Baerenbaum MR (1991). Glucosinolates: Chemistry and ecology.. Herbivores: Their Interactions with Secondary Plant Metabolites. 2nd ed.

[21] Bones AM, Rossiter JT (2006). The enzymic and chemically induced decomposition of glucosinolates.. Phytochemistry.

[22] Lambrix V, Reichelt M, Mitchell-Olds T, Kliebenstein D, Gershenzon J (2001). The Arabidopsis epithiospecifier protein promotes the hydrolysis of glucosinolates to nitriles and influences *Trichoplusia ni* herbivory.. Plant Cell.

[23] Zhang Z-Y, Ober JA, Kliebenstein DJ (2006). The gene controlling the quantitative trait locus *EPITHIOSPECIFIER MODIFIER1* alters glucosinolate hydrolysis and insect resistance in Arabidopsis.. Plant Cell.

[24] Barth C, Jander G (2006). Arabidopsis myrosinases *TGG1* and *TGG2* have redundant function in glucosinolate breakdown and insect defense.. Plant J.

[25] Burow M, Muller R, Gershenzon J, Wittstock U (2006). Altered glucosinolate hydrolysis in genetically engineered Arabidopsis thaliana and its influence on the larval development of Spodoptera littoralis.. J Chem Ecol.

[26] McNaughton SA, Marks GC (2003). Development of a food composition database for the estimation of dietary intakes of glucosinolates, the biologically active constituents of cruciferous vegetables.. Br J Nutr.

[27] Zabala MD, Grant M, Bones AM, Bennett R, Lim YS (2005). Characterisation of recombinant epithiospecifier protein and its over-expression in *Arabidopsis thaliana*.. Phytochemistry.

[28] Foo HL, Gronning LM, Goodenough L, Bones AM, Danielsen B-E (2000). Purification and characterisation of epithiospecifier protein from *Brassica napus*: Enzymic intramolecular sulphur addition within alkenyl thiohydroximates derived from alkenyl glucosinolate hydrolysis.. FEBS Lett.

[29] Bernardi R, Negri A, Ronchi S, Palmieri S (2000). Isolation of the epithiospecifier protein from oil-rape (*Brassica napus* ssp. *oleifera*) seed and its characterization.. FEBS Lett.

[30] Burow M, Rice M, Hause B, Wittstock U, Gershenzon J (2007). Cell- and tissue-specific localization and regulation of the epithiospecifier protein in *Arabidopsis thaliana*.. Plant Mol Biol.

[31] Burow M, Zhang Z-Y, Ober JA, Lambrix VM, Wittstock U (2007). *ESP* and *ESM1* mediate Indol-3-acetonitrile production from Indol-3-ylmethyl glucosinolate in Arabidopsis.. Phytochemistry.

[32] Wentzell AM, Kliebenstein DJ (2008). Genotype, age, tissue, and environment regulate the structural outcome of glucosinolate activation.. Plant Physiol.

[33] Loudet O, Chaillou S, Camilleri C, Bouchez D, Daniel-Vedele F (2002). Bay-0×Shahdara recombinant inbred line population: a powerful tool for the genetic dissection of complex traits in Arabidopsis.. Theor Appl Genet.

[34] Boyes DC, Zayed AM, Ascenzi R, McCaskill AJ, Hoffman NE (2001). Growth stage-based phenotypic analysis of arabidopsis: A model for high throughput functional genomics in plants.. Plant Cell.

[35] Rowe HC, Hansen BG, Halkier BA, Kliebenstein DJ (2008). Biochemical networks and epistasis shape the *Arabidopsis thaliana* metabolome.. Plant Cell.

[36] Bidart-Bouzat MG, Kliebenstein DJ (2008). Differential levels of insect herbivory in the field associated with genotypic variation in glucosinolates in Arabidopsis thaliana.. J Chem Ecol.

[37] West MAL, Kim K, Kliebenstein DJ, van Leeuwen H, Michelmore RW (2007). Global eQTL mapping reveals the complex genetic architecture of transcript level variation in Arabidopsis.. Genetics.

[38] Wentzell AM, Rowe HC, Hansen BG, Ticconi C, Halkier BA (2007). Linking metabolic QTL with network and *cis*-eQTL controlling biosynthetic pathways.. PLoS Genetics.

[39] Kliebenstein DJ, Kroymann J, Brown P, Figuth A, Pedersen D (2001). Genetic control of natural variation in *Arabidopsis thaliana* glucosinolate accumulation.. Plant Physiol.

[40] Mithen R, Clarke J, Lister C, Dean C (1995). Genetics of aliphatic glucosinolates .III. Side-chain structure of aliphatic glucosinolates in *Arabidopsis thaliana*.. Heredity.

[41] Jander G, Cui J, Nhan B, Pierce NE, Ausubel FM (2001). The TASTY locus on chromosome 1 of Arabidopsis affects feeding of the insect herbivore *Trichoplusia ni*.. Plant Physiol.

[42] Kliebenstein D, Lambrix V, Reichelt M, Gershenzon J, Mitchell-Olds T (2001). Gene duplication and the diversification of secondary metabolism: side chain modification of glucosinolates in *Arabidopsis thaliana*.. Plant Cell.

[43] Kroymann J, Textor S, Tokuhisa JG, Falk KL, Bartram S (2001). A gene controlling variation in Arabidopsis glucosinolate composition is part of the methionine chain elongation pathway.. Plant Physiol.

[44] Kliebenstein DJ (2008). A role for gene duplication and natural variation of gene expression in the evolution of metabolism.. PLoS ONE.

[45] Matusheski NV, Swarup R, Juvik JA, Mithen R, Bennett M (2006). Epithiospecifier protein from broccoli (*Brassica oleracea* L. ssp *italica*) inhibits formation of the anticancer agent sulforaphane.. J Agric Food Chem.

[46] Jeffery EH, Brown AF, Kurilich AC, Keck AS, Matusheski N (2003). Variation in content of bioactive components in broccoli.. J Food Comp Anal.

[47] Palaniswamy UR, McAvoy RJ, Bible BB, Stuart JD (2003). Ontogenic variations of ascorbic acid and phenethyl isothiocyanate concentrations in watercress (Nasturtium officinale R.Br.) leaves.. J Agric Food Chem.

[48] Charron CS, Saxton AM, Sams CE (2005). Relationship of climate and genotype to seasonal variation in the glucosinolate-myrosinase system. II. Myrosinase activity in ten cultivars of Brassica oleracea grown in fall and spring seasons.. J Sci Food Agric.

[49] Walsh JB (1995). How Often Do Duplicated Genes Evolve New Functions.. Genetics.

[50] Lynch M, Conery JS (2000). The evolutionary fate and consequences of duplicate genes.. Science.

[51] Lynch M, Force A (2000). The probability of duplicate gene preservation by subfunctionalization.. Genetics.

[52] Somorjai IML, Danzmann RG, Ferguson MM (2003). Distribution of temperature tolerance quantitative trait loci in Arctic charr (Salvelinus alpinus) and inferred homologies in rainbow trout (Oncorhynchus mykiss).. Genetics.

[53] Axelsson T, Shavorskaya O, Lagercrantz U (2001). Multiple flowering time QTLs within several Brassica species could be the result of duplicated copies of one ancestral gene.. Genome.

[54] Loudet O, Gaudon V, Trubuil A, Daniel-Vedele F (2005). Quantitative trait loci controlling root growth and architecture in *Arabidopsis thaliana* confirmed by heterogeneous inbred family.. Theor Appl Genet.

[55] Spencer GF, Daxenbichler ME (1980). Gas chromatography– mass spectrometry of nitriles, isothiocyanates and oxazolidinethiones derived from cruciferous glucosinolates.. J Sci Food Agric.

[56] Zeng Z-B, Kao C-H, Basten CJ (1999). Estimating the genetic architecture of quantitative traits.. Genet Res.

[57] Basten CJ, Weir BS, Zeng Z-B (1999). QTL Cartographer, Version 1.13.

[58] Wang S, Basten CJ, Zeng Z-B (2006). Windows QTL Cartographer 2.5.

[59] Churchill GA, Doerge RW (1994). Empirical Threshold Values For Quantitative Trait Mapping.. Genetics.

[60] Doerge RW, Churchill GA (1996). Permutation tests for multiple loci affecting a quantitative character.. Genetics.

[61] Takechi K, Sakamoto W, Utsugi S, Murata M, Motoyoshi F (1999). Characterization of a flower-specific gene encoding a putative myrosinase binding protein in Arabidopsis thaliana.. Plant Cell Physiol.

[62] Sønderby IE, Hansen BG, Bjarnholt N, Ticconi C, Halkier BA (2007). A systems biology approach identifies a R2R3 MYB gene subfamily with distinct and overlapping functions in regulation of aliphatic glucosinolates.. PLoS ONE.

[63] Hansen BG, Kliebenstein DJ, Halkier BA (2007). Identification of a flavin-monooxygenase as the S-oxygenating enzyme in aliphatic glucosinolate biosynthesis in Arabidopsis.. Plant J.

