# Supplementary figures and images for: Genetic Networks Controlling Structural Outcome of Glucosinolate Activation across Development
Source: PLoS Genet. 2008 Oct 24;4(10):e1000234. doi: 10.1371/journal.pgen.1000234 (PMC2565841; doi:10.1371/journal.pgen.1000234)

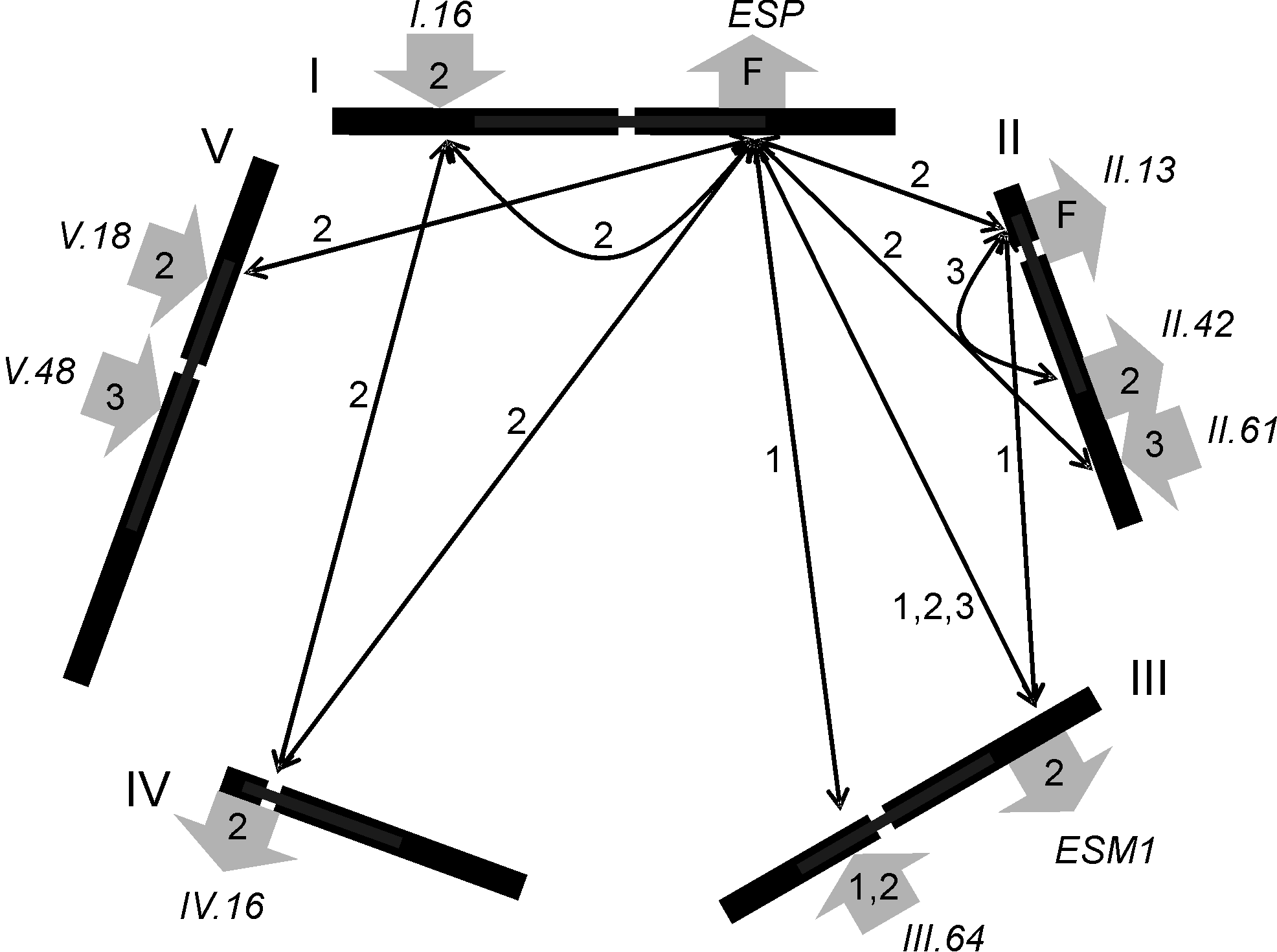

Supplement: Figure S1 — QTLs Controlling the Isothiocyanate Outcome of Glucosinolate Activation. The five Arabidopsis chromosomes are depicted as lines in a pentagonal layout with roman numerals placed at the 0 cM position for each chromosome. Arrows to the outside of each chromosome show the position of the identified QTLs affecting isothiocyanate production. Inside each arrow, a number 1 indicates that the QTL was detected at 30 DPG, 2 for 35 DPG, and 3 for 42 DPG. Arrows for loci with positive allele substitution values for Bay-0 point away from the chromosomes while arrows for QTL with negative allele substitution values point inward. The QTLs are named according to the nomenclature used in the text. Significant epistatic interactions are illustrated with arrows inside the pentagon connecting the interacting loci and numbered to indicate the DPG at which the interaction was detected. (2.90 MB TIF) [file pgen.1000234.s001.tif]
